# Integrated Phytochemical, Anthocyanin, Organic Acid and Colour Profiling of Plant-Derived Syrups as Potential Natural Food Ingredients

**DOI:** 10.3390/foods15142500

**Published:** 2026-07-15

**Authors:** Ingmars Cinkmanis, Fredijs Dimins, Ingrīda Augšpole, Sanita Vucane

**Affiliations:** Faculty of Agriculture and Food Technology, Latvia University of Life Sciences and Technologies, Lielā Street 2, LV-3001 Jelgava, Latvia; ingrida.augspole@lbtu.lv (I.A.); sanita.vucane@lbtu.lv (S.V.)

**Keywords:** plant-derived syrups, bioactive compounds, phenolic compounds, flavonoids, anthocyanins, organic acids, HPLC, CIELAB colour, chemometrics, principal component analysis

## Abstract

Plant-derived syrups are traditional sugar-rich products that may serve as potential natural food ingredients, but their quality-related characteristics depend on raw material composition, acidity, phenolic constituents, pigments and colour attributes. This study characterised 15 plant-derived syrup products prepared from botanical and fungal raw materials collected in Latvia during the 2025 vegetation season. The syrups were analysed for pH, total soluble solids, titratable acidity, total phenolic content (TPC), total flavonoid content (TFC), total anthocyanin content (TAC) determined spectrophotometrically, smartphone-based CIELAB colour parameters and organic acid content by HPLC-DAD. Data were evaluated using correlation analysis, PCA and K-means clustering as exploratory tools. The syrup products differed in acidity, phenolic and flavonoid indices, TAC, colour characteristics and organic acid profiles. Fruit-derived syrups showed the widest variation in titratable acidity, while total soluble solids showed no significant linear association with titratable acidity. TPC correlated strongly with TFC in samples with quantifiable TFC values, whereas TAC was associated more closely with reduced lightness than with increased redness. Organic acid profiles provided additional product-level differentiation. Overall, the integrated analytical approach provides a compositional basis for the further evaluation of plant-derived syrups as potential natural food ingredients.

## 1. Introduction

Plant-derived syrups represent a traditional category of food products that combine botanical raw materials, concentrated sugar matrices and naturally occurring plant constituents. In recent years, interest in medicinal and edible plants and their derived products has increased because they are recognised as important sources of phenolic compounds, flavonoids, organic acids, pigments and other secondary metabolites. The World Health Organization has reported that a considerable proportion of the global population uses plant-based products in primary health care, highlighting their continued importance in nutrition, traditional practice and food product development [1]. Among such products, herbal and fruit syrups are valued for their colour, acidity, sweetness, ease of consumption and potential use as natural food ingredients [2,3].

The composition and quality characteristics of plant-derived syrups depend on both the raw material and the preparation conditions used to obtain the final syrup product. Fruits, berries, flowers, young shoots and bark differ considerably in their phytochemical composition, particularly in the content of phenolic compounds, flavonoids, anthocyanins and organic acids. These compounds contribute to antioxidant-related properties, acidity, flavour, colour formation and overall product quality. Phenolic compounds and flavonoids are widely recognised as important natural antioxidants because of their ability to participate in redox reactions and scavenge free radicals [4,5]. However, compositional indices alone do not confirm functional performance, and further antioxidant activity, bioaccessibility and biological activity studies are required before functional food effects can be established [6,7].

Organic acids are another important group of compounds in fruit and herbal syrups. They influence pH, titratable acidity, taste, aroma, microbial stability and shelf-life, and therefore play a central role in the physicochemical quality of syrup products [8]. Their composition and concentration may vary substantially depending on plant species, maturity stage, environmental conditions and processing. Soluble solids content is also an important quality parameter in syrup production because it reflects the sugar-rich nature of the matrix and affects sweetness, viscosity and preservation [9]. The combined interpretation of acidity and soluble solids is therefore relevant for describing the technological and quality-related characteristics of final syrup products.

Colour is one of the most important visual quality attributes of plant-derived syrups, affecting consumer perception, product differentiation and potential application as natural food ingredients [10]. Anthocyanins are among the principal water-soluble pigments responsible for red, purple and blue colouration in many plant materials, especially fruits and berries, and their stability is strongly influenced by pH and food matrix conditions [11,12]. Instrumental colour assessment using the CIELAB system enables the objective description of lightness (L*), redness-greenness (a*) and yellowness-blueness (b*) and is widely applied in food colour evaluation [10]. Associations between colour parameters, phenolic compounds and antioxidant-related properties have also been reported in fruit matrices, supporting the relevance of combining instrumental colour and phytochemical data [13,14]. However, in herbal and fruit syrup research, colour parameters and total anthocyanin content have rarely been interpreted together with phenolic indices, acidity and organic acid composition within one comparative dataset.

Previous studies on Latvian herbal and fruit syrups have provided important information on selected groups of quality-related compounds. Cinkmanis et al. investigated organic acids, pH and dry matter in a range of herbal and fruit syrups using high-performance liquid chromatography [3], whereas Dimiņš and Augšpole characterised total phenolic content, total flavonoid content and antiradical activity in herbal syrups [2]. These studies demonstrated that Latvian plant-derived syrups differ markedly in organic acid composition and antioxidant-related parameters. Nevertheless, these earlier investigations considered individual analytical groups separately and did not integrate acidity, soluble solids, phenolic and flavonoid indices, spectrophotometric total anthocyanin content, smartphone-based CIELAB colour parameters and organic acid composition within a single comparative product-level dataset.

The present research addresses this gap by providing an integrated characterisation of 15 plant-derived syrup products prepared from raw materials collected in Latvia during the 2025 vegetation season. By combining physicochemical measurements, phenolic and flavonoid indices, spectrophotometric TAC, smartphone-based CIELAB colour analysis and HPLC-DAD-based organic acid determination, this study enables final syrup products to be compared according to both compositional and visual quality-related attributes.

The aim of this research was to evaluate how the combined analytical dataset differentiates selected plant-derived syrup products and to provide a compositional basis for their potential selection as natural food ingredients.

## 2. Materials and Methods

### 2.1. Research Design and Experimental Framework

This research was designed as a comparative analytical characterisation of 15 plant-derived syrup products prepared from botanically diverse raw materials collected in Latvia during the 2025 vegetation season. The primary comparison factor was the type of raw material used for syrup preparation, while the secondary categorical factor was the raw material matrix group, including fruits, berries, flowers, young shoots, bark and other traditionally used natural raw materials. These matrix groups were used to organise the syrup products according to the morphological and compositional characteristics of the raw materials, rather than to establish definitive botanical matrix effects.

To improve comparability among the prepared syrup products, the main processing conditions were standardised where applicable. The controlled factors included raw material collection within the same vegetation season, comparable post-harvest handling, the use of deionised water as the extraction medium, a fixed raw-material-to-water ratio, matrix-adapted thermal extraction, standardised sucrose addition, hot filling into sterilised glass containers and storage under controlled dark conditions until analysis. Because the raw materials differed in structure, extractability and initial moisture content, the comparison was interpreted at the level of final syrup products rather than as a dry-matter-based comparison of the original raw materials.

The comparative framework was based on evaluating compositional, physicochemical and colour-related differences among the final syrup products. The response variables included pH, total soluble solids, titratable acidity, total phenolic content, total flavonoid content, total anthocyanin content, CIELAB colour parameters and organic acid composition. Results were expressed on a final syrup mass basis and therefore describe the prepared syrup products as analysed, not the concentration of compounds in the original raw materials on a dry-matter basis.

For each syrup type, one prepared syrup batch was analysed. Analytical measurements were performed in triplicate from the same prepared syrup batch, and the results are reported as mean ± SD of technical replicates. Therefore, the reported variability reflects analytical repeatability rather than biological or independent batch-to-batch variability. This design enabled exploratory product-level comparison of syrup composition across different raw material matrices while reducing variability related to season, post-harvest handling, extraction medium, sucrose addition and storage conditions.

### 2.2. Plant Material Collection and Syrup Samples

A total of 15 syrup types were prepared from diverse traditionally used botanical and fungal raw materials collected in Latvia during the 2025 vegetation season. The raw materials included fruits, flowers, young shoots, cones, bark and fungal material. Fresh raw materials were harvested at appropriate maturity or developmental stages and transported to the laboratory under ambient conditions on the day of collection.

Raw materials were taxonomically identified according to standard botanical and mycological references. For samples identified at genus level, such as *Crataegus* spp. and *Betula* spp., genus-level classification was retained because species-level identification was not required for the product-level comparative design of the research. The analysed samples were treated as final syrup products prepared from defined raw material sources, rather than as dry-matter-normalised raw material extracts.

Detailed information on syrup type, botanical origin and harvesting location is presented in Table 1.

All syrup preparation and analytical work was carried out in the laboratories of the Institute of Food Science, Faculty of Agriculture and Food Technology, Latvia University of Life Sciences and Technologies (LBTU), Jelgava, Latvia.

### 2.3. Preparation of Plant-Derived Syrups

Raw materials were processed according to their morphological characteristics. Fresh fruits and fruit-like raw materials were washed with deionised water and mechanically crushed or pressed to facilitate cell disruption and juice release. More rigid raw materials, including cones, bark and chaga material, were finely chopped to an approximate particle size of 2–5 mm to increase the extraction surface area.

Extraction was performed using deionised water at a fixed raw-material-to-water ratio of 1:5 (*w*/*v*). The raw material was immersed in deionised water and heated to boiling temperature (100 ± 2 °C) using a thermostatically controlled heating plate. Extraction conditions were adapted according to matrix type. Soft plant materials, including flowers and other delicate tissues, were heated for 5 min and then macerated at room temperature (20 ± 2 °C) for 12 h. Dense materials, including fruits, cones, bark and chaga material, were subjected to continuous boiling for 30–60 min in covered extraction vessels. After thermal treatment, the material was further homogenised using a sterile pestle to enhance extraction efficiency.

The pH of the extraction medium was monitored during processing; however, no artificial pH adjustment or buffering step was applied during syrup preparation. To minimise oxidative degradation, extraction vessels were covered and exposure to air was reduced. After extraction, the samples were rapidly cooled to room temperature. The extracts were filtered through a double layer of muslin cloth followed by filtration through a fine sieve with a mesh size below 0.5 mm. The filtrate volume was recorded for each syrup preparation.

Sucrose was added at a concentration of 1.2 kg per 1 L of measured filtrate, with an allowed variation of ±2%. The mixture was heated at 85–90 °C under constant stirring until complete sucrose dissolution and syrup consistency were achieved. The target soluble solids content of the final syrups was approximately 65–70 °Brix; however, the final total soluble solids were measured analytically for each syrup and were allowed to vary depending on the soluble compounds extracted from each raw material matrix and minor concentration effects during heating. Foam formed during heating was removed manually. The final syrups were hot-filled into sterilised glass containers at temperatures above 85 °C, hermetically sealed and cooled to room temperature to ensure vacuum formation.

All syrup samples were stored at 15 ± 2 °C in the dark until analysis.

### 2.4. Chemicals, Reagents and Standards

All chemicals and reagents used in this research were of analytical grade or higher. Folin–Ciocalteu reagent, gallic acid, quercetin, aluminium chloride (AlCl_3_), sodium carbonate (Na_2_CO_3_), sodium hydroxide (NaOH), phenolphthalein, hydrochloric acid (HCl), ethanol and other reagents used for spectrophotometric assays were obtained from Sigma-Aldrich Chemie GmbH (Steinheim, Germany), Merck KGaA (Darmstadt, Germany) or Fluka Chemie GmbH (Buchs, Switzerland), according to reagent availability. Methanol and phosphoric acid (H_3_PO_4_) used for chromatographic analysis were of HPLC grade and were obtained from Merck KGaA (Darmstadt, Germany).

Organic acid standards, including oxalic acid, tartaric acid, quinic acid, malic acid, ascorbic acid, citric acid, fumaric acid and succinic acid, were purchased from Sigma-Aldrich Chemie GmbH (Steinheim, Germany) or Fluka Chemie GmbH (Buchs, Switzerland). Gallic acid and quercetin were used as calibration standards for total phenolic and total flavonoid content, respectively.

Deionised water was used throughout the research for raw material washing, syrup extraction, sample dilution, preparation of analytical solutions and preparation of calibration standards. Standard stock and working solutions were prepared using the solvent systems specified for each analytical method. Working standards were prepared freshly before analysis or stored under conditions appropriate to the stability of each compound in order to minimise degradation.

### 2.5. Determination of pH, Total Soluble Solids and Titratable Acidity

The pH of the syrup samples was determined potentiometrically using a WTW inoLab pH metre (WTW GmbH, Weilheim, Germany). Before analysis, the instrument was calibrated with standard buffer solutions at pH 4.01, 7.00 and 10.01. Measurements were performed at 20 ± 1 °C, and the electrode was rinsed with deionised water and gently dried between measurements.

Total soluble solids (TSS) were determined using a KERN ORL 94LM digital refractometer (Kern & Sohn GmbH, Balingen, Germany) with an accuracy of ±0.1 °Brix and automatic temperature compensation. Before measurement, the refractometer was calibrated with deionised water. Syrup samples were placed directly on the prism surface, and the prism was cleaned with deionised water and dried with lint-free tissue between measurements. The results were expressed as °Brix. All measurements were performed at 20 ± 1 °C.

Titratable acidity was determined using a volumetric titration method adapted from AOAC Official Method 942.15 for titratable acidity in fruit products and Paul et al. [15,16], with modifications for syrup matrices. A 10.00 ± 0.01 g syrup sample was dissolved in 100 mL of deionised water and homogenised. When necessary, the solution was filtered before titration. The prepared solution was titrated with 0.1 mol L^−1^ NaOH using phenolphthalein as an indicator until a stable pale pink colour persisted for approximately 30 s, corresponding to an endpoint close to pH 8.2.

Titratable acidity was expressed as mg citric acid equivalents 100 g^−1^ syrup and calculated using Equation (1):

(1) $$TA=\frac{V\;\times \;C\;\times \;64.04\;\times \;100}{m}$$

where:

*TA*—titratable acidity expressed as mg citric acid equivalents per 100 g of syrup;

*V*—volume of NaOH used for titration (mL);

*C*—concentration of NaOH (mol L^−1^);

64.04—equivalent mass of citric acid (mg mmol^−1^);

100—conversion factor for expression per 100 g of syrup;

*m*—mass of the syrup sample (g).

### 2.6. Determination of Total Phenolic Content

Total phenolic content (TPC) was determined using the Folin–Ciocalteu colorimetric method adapted from Kaškoniene, [17]. A syrup sample of 5.00 ± 0.01 g was diluted to 50 mL with deionised water, homogenised and filtered through a 0.45 µm hydrophilic syringe filter before analysis. Where necessary, the filtered extract was further diluted with deionised water to keep the absorbance within the calibration range.

An aliquot of the filtered extract (0.5 mL) was mixed with 2.5 mL of 0.2 M Folin–Ciocalteu reagent. After 5 min, 2.0 mL of sodium carbonate solution (75 g L^−1^) was added. The reaction mixture was incubated at room temperature (20 ± 2 °C) for 120 min in the dark. Absorbance was measured at 760 nm using a UV-1900i UV-Vis spectrophotometer (Shimadzu Corporation, Kyoto, Japan) against a reagent blank.

Gallic acid was used as the calibration standard, and the calibration curve was prepared in the concentration range of 20–200 mg L^−1^. Linearity was accepted when the coefficient of determination was R^2^ ≥ 0.999. Results were expressed as mg gallic acid equivalents 100 g^−1^ syrup (mg GAE 100 g^−1^).

### 2.7. Determination of Total Flavonoid Content

Total flavonoid content (TFC) was determined using the aluminium chloride colorimetric method adapted from Meda [18]. A syrup sample of 1.00 ± 0.01 g was mixed with 50 mL of methanol, homogenised and filtered through a 0.45 µm hydrophilic syringe filter before analysis. A fixed dilution protocol was applied to all samples.

An aliquot of the prepared extract (5.0 mL) was mixed with 5.0 mL of 2% aluminium chloride solution in methanol. The reaction mixture was incubated at room temperature (20 ± 2 °C) for 10 min. Absorbance was measured at 415 nm using a UV-1900i UV-Vis spectrophotometer (Shimadzu Corporation, Kyoto, Japan). The blank consisted of the corresponding sample solution mixed with methanol without aluminium chloride, thereby accounting for sample background absorbance under the applied assay conditions.

Quercetin was used as the calibration standard, and the calibration curve was prepared in the concentration range of 10–50 mg L^−1^. Linearity was accepted when the coefficient of determination was R^2^ ≥ 0.999. Results were expressed as mg quercetin equivalents 100 g^−1^ syrup (mg QE 100 g^−1^).

### 2.8. Determination of Total Anthocyanin Content

Total anthocyanin content (TAC) was determined using an acidified ethanol spectrophotometric method adapted from Kerch et al. [19] and Ozola and Kampuse [20], with modifications for plant-derived syrup matrices.

A syrup sample of 0.50 ± 0.01 g was accurately weighed into a centrifuge tube and extracted with 10.0 mL of ethanol:1.5 M HCl solution (85:15, *v*/*v*). The mixture was homogenised for 1 min and extracted for 30 min at room temperature (20 ± 2 °C) in the dark. The samples were then centrifuged at 4000 rpm for 10 min, and the supernatant was collected. When necessary, the extract was diluted with the extraction solvent to obtain absorbance values within the recommended absorbance range.

Absorbance was measured immediately after extraction using a UV-1900i UV-Vis spectrophotometer (Shimadzu Corporation, Kyoto, Japan) at 540 nm. The extraction solvent was used as a reagent blank.

TAC was calculated according to the spectrophotometric equation used in the acidified ethanol method, based on absorbance, extraction volume, dilution factor and sample mass. Results were expressed as mg 100 g^−1^ syrup and calculated according to Equation (2):

(2) $$TAC=\frac{A\;\times \;V\;\times \;DF\;\times \;1000}{980\;\times \;m}$$

where:

*TAC*—total anthocyanin content expressed as mg 100 g^−1^ syrup;

*A*—absorbance of the extract measured at 540 nm;

*V*—extraction volume (mL);

*DF*—dilution factor;

1000—conversion factor used in the applied spectrophotometric method;

980—method coefficient used for anthocyanin calculation under the applied acidified ethanol method;

*m*—mass of the syrup sample (g).

### 2.9. Instrumental Colour Analysis Using Smartphone-Based DiColorimetry and the CIELAB System

Colour analysis of plant-derived syrups was performed using a smartphone-based imaging system and the DiColorimetry application. Image acquisition was carried out in a portable photographic light box (Puluz Technology Ltd., Shenzhen, China) to minimise the influence of ambient light and ensure reproducible illumination. The light box was equipped with 40 type-2835 LED light sources and operated using a 5 V USB power supply. The system provided stable illumination with nominal colour temperatures of 3200 K and 6500 K and a colour rendering index above 82.

Syrup samples were transferred into disposable 2.5 mL polystyrene macro cuvettes (BrandTech Scientific, Essex, CT, USA) and placed inside the illuminated chamber against a uniform white background. Images were acquired using a Motorola Edge 40 Neo 5G smartphone (Motorola Mobility, Beijing, China). The smartphone was positioned horizontally at a fixed distance of 12 cm from the front opening of the light box, and the same imaging geometry was maintained for all samples. A ColorChecker^®^ Classic reference card (X-Rite, Inc., Grand Rapids, MI, USA) was used to verify colour consistency during image acquisition. A representative screenshot of the DiColorimetry interface used for image acquisition and region selection is provided as Figure S1 in the Supplementary Materials.

Images were captured using the integrated main camera of the smartphone and stored in JPEG format at a resolution of 3072 × 4096 pixels. The recorded acquisition parameters were aperture f/1.8, exposure time 1/50 s, ISO 260 and focal length 5.56 mm. Colour extraction and processing were performed using DiColorimetry version 1.2, a custom Android application developed for smartphone-based image analysis and colour coordinate extraction [21]. The application was used to extract colour information from selected regions of interest and to obtain colour coordinates in the RGB and CIE Lab* colour spaces.

For each syrup sample, the region of interest was selected from the central homogeneous area of the cuvette image, avoiding edges, reflections and air bubbles. The CIELAB parameters L*, a* and b* were recorded, where L* represents lightness, a* represents the green-red axis and b* represents the blue–yellow axis.

### 2.10. Determination of Organic Acids by HPLC

Organic acid composition was determined by high-performance liquid chromatography with diode array detection (HPLC-DAD), using a method adapted from a previously published liquid chromatographic procedure for organic acid analysis [22].

A syrup sample of 1.00 ± 0.01 g was accurately weighed and diluted with deionised water to a final volume of 10.0 mL. The mixture was homogenised thoroughly and filtered through a 0.45 µm hydrophilic syringe filter before chromatographic analysis.

A mixed organic acid stock standard solution was prepared by accurately weighing 0.100 ± 0.001 g each of oxalic acid, tartaric acid, quinic acid, malic acid, ascorbic acid, citric acid and succinic acid, and 0.020 ± 0.001 g of fumaric acid into a 50.00 ± 0.05 mL volumetric flask. The compounds were dissolved in deionised water and diluted to the mark. Working standard solutions were prepared by serial dilution to obtain at least five concentration levels for calibration.

Chromatographic analysis was performed using a Shimadzu LC–20 Prominence HPLC system equipped with a diode array detector (SPD-M20A; Shimadzu Corporation, Kyoto, Japan). Separation was carried out on a YMC C18 analytical column (4.6 × 250 mm, 5 µm; YMC Co., Ltd., Kyoto, Japan). The column temperature was maintained at 35 °C. The mobile phase consisted of 0.1% phosphoric acid aqueous solution under isocratic elution conditions. The flow rate was 1.0 mL min^−1^, the injection volume was 10 µL and the total run time was 20 min.

**Figure 1:**
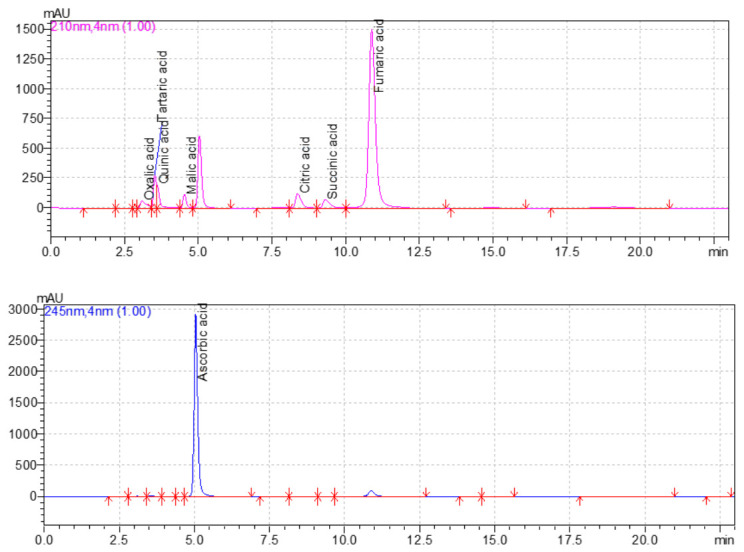
Chromatogram of the calibration solution of the organic acids.

Detection was performed at 210 nm for oxalic, tartaric, quinic, malic, citric, fumaric and succinic acids, and at 245 nm for ascorbic acid. A representative chromatogram of the organic acid calibration solution is shown in Figure 1.

Organic acids were identified by comparing retention times and UV spectra with those of the corresponding analytical standards analysed under the same chromatographic conditions. Quantification was performed using external calibration curves prepared from mixed organic-acid-standard solutions. Peaks were reported as not detected when no identifiable peak corresponding to the retention time and UV spectral characteristics of the analytical standard was observed under the applied chromatographic conditions.

The content of individual organic acids was expressed as mg 100 g^−1^ syrup and calculated using Equation (3):

(3) $$OA=\frac{C\;\times \;V\;\times \;100}{m}$$

where:

*OA*—is the content of an individual organic acid, expressed as mg 100 g^−1^ of syrup;

*C*—concentration of the corresponding organic acid detected in the prepared sample solution by HPLC-DAD (mg L^−1^);

*V*—final volume of the sample solution (L);

*m*—mass of the syrup sample (g).

### 2.11. Analytical Quality Control and Statistical Analysis

Analytical quality control was ensured by the use of external calibration standards, reagent blanks and replicate measurements. Calibration curves were prepared for gallic acid, quercetin and individual organic acid standards using at least five concentration levels within the expected analytical range. Calibration curves were constructed by plotting analytical response against standard concentration, and linearity was accepted when the coefficient of determination was R^2^ ≥ 0.999. Reagent blanks were included in each analytical batch. Blank values were subtracted from spectrophotometric absorbance readings where appropriate, while chromatographic blanks were used to verify the absence of interfering peaks and carry-over under the applied HPLC-DAD conditions.

For each syrup type, one prepared syrup batch was analysed. Analytical measurements were performed in triplicate from the same prepared syrup batch, and the results were expressed as mean ± standard deviation (SD) of technical replicates. Technical replicates were used to estimate analytical repeatability and were not treated as independent biological replicates. Statistical and chemometric analyses were performed using Python 3.11.3. The significance level was set at *p* < 0.05. All statistical analyses were performed using the mean value calculated from technical replicate measurements for each of the 15 syrup types. Therefore, the statistical outputs were interpreted as exploratory product-level associations rather than confirmatory biological or batch-to-batch effects.

Pearson correlation analysis and simple linear regression were used to evaluate selected linear associations between physicochemical, phytochemical and colour-related variables. Pearson correlation coefficients (r), *p*-values and coefficients of determination (R^2^) were reported for regression-based models. Because of the limited number of syrup products, these analyses were interpreted as exploratory associations.

Spearman’s rank correlation analysis was performed to evaluate exploratory monotonic associations between organic acids, phenolic indices and CIELAB colour parameters. Spearman correlation coefficients were visualised using a correlation heatmap to identify positive and negative associations between these variables. Values reported as n.d. for organic acids were treated as not detected under the applied chromatographic conditions for exploratory multivariate analysis. Correlations involving variables with non-quantifiable values were calculated using the available quantifiable data. Because multiple pairwise correlations were examined, the heatmap was interpreted as an exploratory pattern-recognition tool rather than as confirmatory statistical evidence.

Principal component analysis (PCA) was applied to explore multivariate patterns among syrup samples and to evaluate the contribution of individual variables to sample differentiation. Prior to PCA, variables were autoscaled by standardisation to zero mean and unit variance to reduce the influence of different measurement scales. PCA scores were used to visualise sample distribution, while PCA loadings were used to interpret variable contributions. PCA was interpreted as an exploratory visualisation method because of the limited number of analysed syrup products.

K-means clustering was applied as an exploratory chemometric approach to identify groups of syrups with similar compositional and colour characteristics. Clustering was performed using PCA score data obtained from autoscaled variables. Candidate cluster numbers from k = 2 to k = 5 were evaluated using silhouette scores, and the final cluster solution was selected by considering both the silhouette score and interpretability of sample distribution in the PCA score space. K-means clustering was performed using a fixed random seed of 42 to ensure reproducibility. Cluster interpretation was based on the distribution of phenolic indices, spectrophotometric TAC, CIELAB colour parameters and organic acid profiles. The resulting clusters were interpreted only as exploratory groupings and not as definitive botanical or functional classifications.

### 2.12. Data Availability and Ethical Statement

The DiColorimetry Android application used for smartphone-based colour analysis is openly accessible through the Zenodo repository under DOI 10.5281/zenodo.17819093. The repository provides the Android installation file together with supporting documentation and technical metadata.

No human participants, human-derived materials or animals were involved in this research. Therefore, ethical approval was not required.

## 3. Results

The analysed syrup products showed marked product-level differences in physicochemical parameters, phenolic and flavonoid indices, spectrophotometric total anthocyanin content, smartphone-based CIELAB colour parameters and organic acid composition. These differences reflect the combined influence of raw material characteristics, matrix-adapted preparation and final syrup composition.

The results are presented according to the main analytical groups: physicochemical parameters, total phenolic content, total flavonoid content, spectrophotometric total anthocyanin content, smartphone-based colour characteristics and HPLC-DAD-determined organic acid composition. Particular attention is given to associations between compositional parameters and visual colour attributes, as these parameters are relevant for the product-level characterisation of plant-derived syrups as potential natural food ingredients.

### 3.1. Physicochemical Parameters

The physicochemical characteristics of the analysed syrup products are presented in Table 2. The syrups showed acidic pH values ranging from 2.60 to 5.40. The lowest pH values were measured for quince fruit and birch bark syrups, whereas the highest pH was observed in lilac flower syrup. Total soluble solids (TSS) ranged from 59.4 to 76.7 °Brix, reflecting the concentrated sugar-rich nature of the final syrup products. Titratable acidity (TA) varied more widely than pH and TSS, ranging from 49.15 to 1347.19 mg CAE 100 g^−1^. The highest TA was observed in quince fruit syrup.

**Figure 2:**
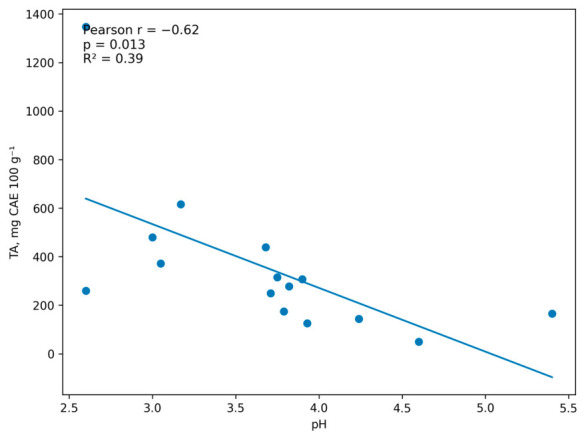
Correlation between pH and titratable acidity (TA) in plant-derived syrups.

The correlation between pH and titratable acidity was evaluated using Pearson correlation and simple linear regression analysis (Figure 2). A statistically significant negative correlation was observed between pH and TA (r = −0.62, *p* = 0.013; R^2^ = 0.39), indicating that syrup products with higher titratable acidity generally showed lower pH values. A sensitivity analysis excluding quince fruit syrup gave a similar pH-TA correlation (r = −0.62, *p* = 0.018; R^2^ = 0.38).

**Figure 3:**
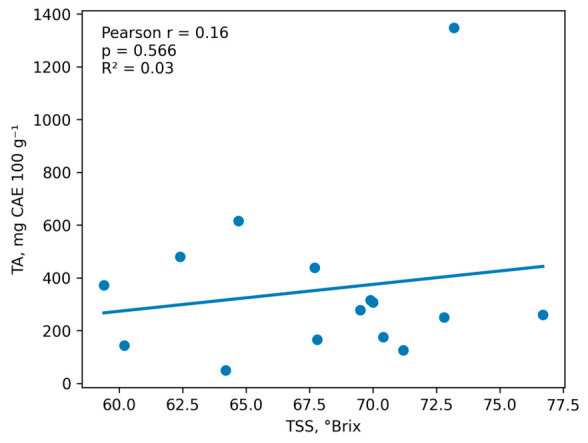
Correlation between total soluble solids (TSS) and titratable acidity (TA) in plant-derived syrups.

The correlation between total soluble solids and titratable acidity was also evaluated using Pearson correlation and simple linear regression analysis (Figure 3). A weak and statistically non-significant correlation was observed between TSS and TA (r = 0.16, *p* = 0.566; R^2^ = 0.03), indicating that no significant linear association was detected between soluble solids and titratable acidity in the analysed syrup products. Excluding quince fruit syrup did not change this interpretation (r = −0.21, *p* = 0.480; R^2^ = 0.04).

**Figure 4:**
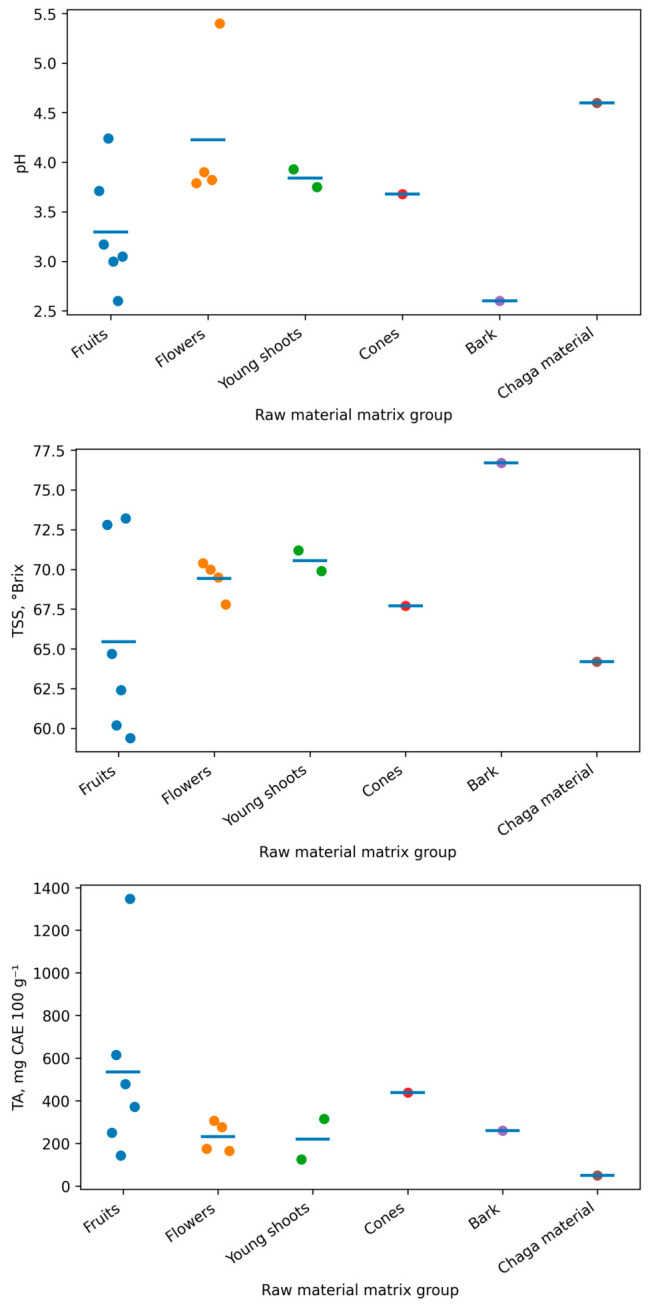
Distribution of physicochemical parameters across raw material matrix groups. Individual points represent syrup products, and horizontal bars indicate group means.

The distribution of physicochemical parameters across raw material matrix groups is shown in Figure 4. Individual-value plots were used because several matrix groups contained only one or two syrup products.

Fruit-derived syrups showed the widest variation in TA, mainly because quince fruit syrup had a markedly higher TA value than the other analysed syrup products. Flower-derived syrups showed a narrower TA range, whereas young shoot syrups showed intermediate acidity-related values. Birch chaga syrup showed the lowest TA value, while birch bark syrup showed low pH despite moderate TA. These matrix-group patterns were descriptive because the groups were small and unbalanced.

TSS showed no significant linear correlation with TA and varied less systematically across matrix groups than acidity-related parameters. Overall, the physicochemical results showed product-level differences in acidity and soluble solids among the analysed syrups, while matrix-group patterns should be interpreted as exploratory descriptive trends rather than statistically confirmed group effects.

### 3.2. Phenolic Composition and Colour Properties of Syrups

The phenolic indices and smartphone-based CIELAB colour characteristics of the analysed syrup products are presented in Table 3. Total phenolic content (TPC) varied markedly among the samples, ranging from 64.87 to 521.64 mg GAE 100 g^−1^. The highest TPC values were observed in meadowsweet flower and rosebay willowherb flower syrups, whereas the lowest values were recorded in birch bark and apple fruit syrups. Total flavonoid content (TFC) also showed substantial variation. Quantifiable TFC values ranged from 3.25 to 62.34 mg QE 100 g^−1^, with the highest values observed in meadowsweet flower and rosebay willowherb flower syrups. In pine cone, spruce young shoot and quince fruit syrups, TFC was below 1 mg QE 100 g^−1^.

Spectrophotometric total anthocyanin content (TAC), determined using the acidified ethanol method at 540 nm, ranged from 0.13 to 3.03 mg 100 g^−1^. The highest TAC values were recorded in pine cone and spruce young shoot syrups, followed by guelder-rose fruit and rowan fruit syrups. Smartphone-based CIELAB colour parameters also differed among samples. Lightness (L*) ranged from 18.590 to 68.171, with darker samples showing lower L* values. The a* coordinate ranged from −2.142 to 22.209, while b* ranged from 1.608 to 51.584.

**Figure 5:**
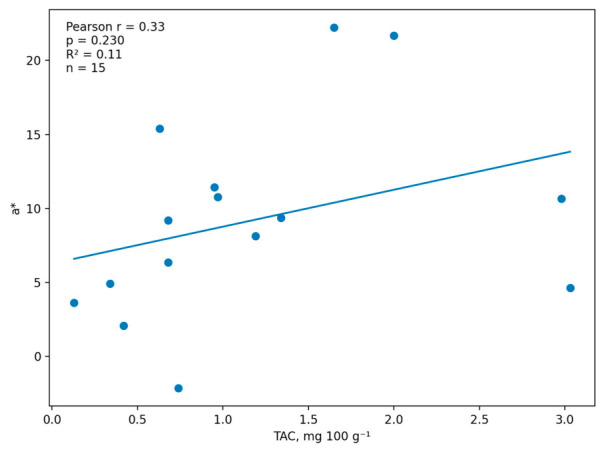
Correlation between spectrophotometric total anthocyanin content (TAC) and a* values in plant-derived syrups.

The correlation between TAC and a* values was evaluated using Pearson correlation and simple linear regression analysis (Figure 5). A weak and statistically non-significant correlation was observed between TAC and a* values (r = 0.33, *p* = 0.230; R^2^ = 0.11; *n* = 15), indicating that TAC was not a strong linear predictor of the red–green colour coordinate in the analysed syrup products.

**Figure 6:**
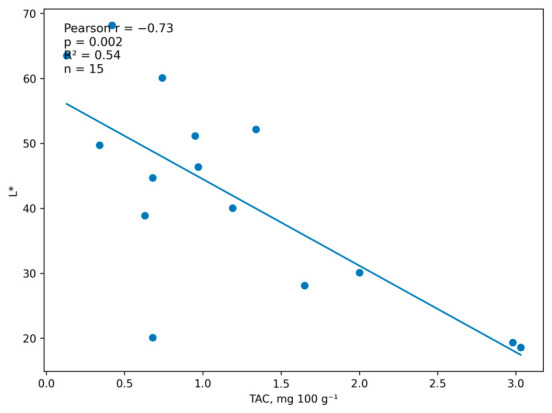
Correlation between spectrophotometric total anthocyanin content (TAC) and L* values in plant-derived syrups.

A statistically significant negative correlation was observed between TAC and L* values (r = −0.73, *p* = 0.002; R^2^ = 0.54; *n* = 15) (Figure 6). Thus, syrup products with higher TAC values generally showed lower lightness values and darker appearance. This association was stronger than the correlation between TAC and a*, indicating that TAC was more closely related to reduced lightness than to redness in the present dataset.

**Figure 7:**
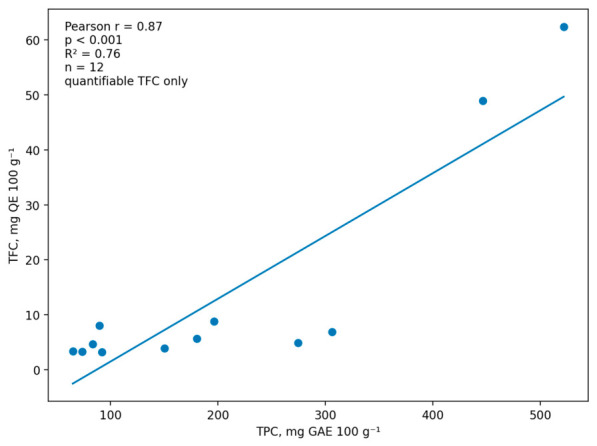
Correlation between total phenolic content (TPC) and total flavonoid content (TFC) in plant-derived syrups with quantifiable TFC values.

The correlation between TPC and TFC was evaluated using only syrup products with quantifiable TFC values. A strong and statistically significant positive correlation was observed between TPC and TFC (r = 0.87, *p* < 0.001; R^2^ = 0.76; *n* = 12) (Figure 7). This indicates that syrup products with higher TPC generally also showed higher quantifiable TFC values.

**Figure 8:**
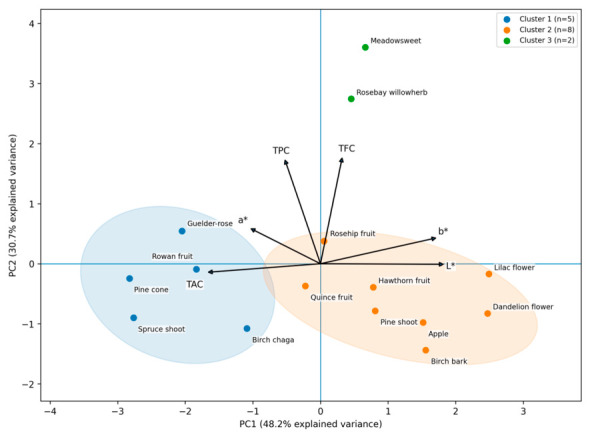
Exploratory PCA biplot of plant-derived syrups based on phenolic indices, spectrophotometric TAC and smartphone-based CIELAB colour parameters.

Principal component analysis (PCA) combined with K-means clustering was applied as an exploratory multivariate visualisation of phenolic indices and smartphone-based CIELAB colour parameters (Figure 8). For PCA only, TFC values reported as <1 mg QE 100 g^−1^ were treated as left-censored values and replaced by 0.5 mg QE 100 g^−1^ to allow inclusion of all 15 syrup products. The first two principal components explained 78.9% of the total variance, with PC1 accounting for 48.2% and PC2 for 30.7%.

In the PCA biplot, points represent syrup products and arrows represent variable loadings. PC1 was mainly associated with colour-related variation. Samples with positive PC1 scores were generally associated with higher L* and b* values, corresponding to lighter and more yellow-coloured syrups, whereas samples with negative PC1 scores were more closely associated with higher TAC values and darker colour characteristics. PC2 was mainly associated with TPC and TFC, separating syrup products with higher phenolic and flavonoid indices from those with lower values.

The PCA biplot showed three exploratory sample groups. Meadowsweet flower and rosebay willowherb flower syrups were positioned in the direction of the TPC and TFC loading vectors. Pine cone, spruce young shoot, rowan fruit and guelder-rose fruit syrups were located closer to the TAC and a* directions and were characterised by darker and/or redder colour attributes. Dandelion flower, lilac flower, apple fruit and birch bark syrups were positioned closer to the L* and b* directions, reflecting lighter and more yellow colour characteristics.

Overall, the combined analysis showed that phenolic indices, spectrophotometric TAC and smartphone-based CIELAB parameters described complementary aspects of syrup product variation. The PCA and clustering results were interpreted as exploratory product-level patterns rather than definitive botanical or functional classifications.

### 3.3. Organic Acid Content

The organic acid content of the analysed syrup products is presented in Table 4. The profiles differed markedly among the 15 syrup products, with oxalic, tartaric, quinic, malic, citric, fumaric, succinic and ascorbic acids detected at different levels depending on the sample. The highest malic acid content was observed in quince fruit syrup, whereas birch chaga syrup showed the highest citric acid content in the present dataset. Quinic acid was particularly abundant in birch chaga and spruce young shoot syrups, while tartaric acid showed higher values in birch chaga and hawthorn fruit syrups. Fumaric acid was detected only at low levels in selected samples. These results show that individual organic acids provide complementary information to titratable acidity and help describe product-level compositional differences among the analysed syrups.

**Figure 9:**
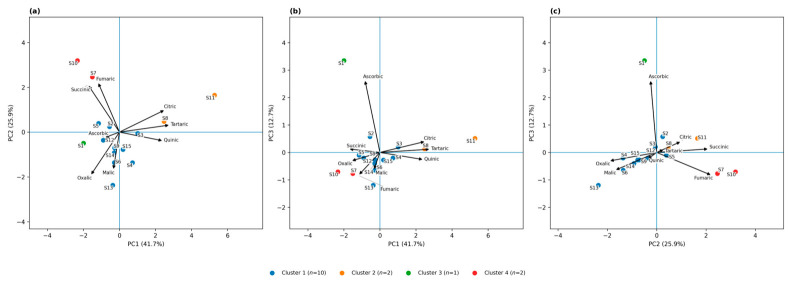
Exploratory PCA biplots of organic acid profiles in plant-derived syrups: (**a**) PC1 versus PC2; (**b**) PC1 versus PC3; and (**c**) PC2 versus PC3. Points represent syrup products, arrows represent organic acid loadings, and colours indicate exploratory K-means clusters. Sample codes: S1, pine cone; S2, rosehip fruit; S3, pine young shoot; S4, spruce young shoot; S5, dandelion flower; S6, rowan fruit; S7, rosebay willowherb flower; S8, hawthorn fruit; S9, apple fruit; S10, meadowsweet flower; S11, birch chaga; S12, lilac flower; S13, quince fruit; S14, guelder-rose fruit; S15, birch bark.

Principal component analysis (PCA) was applied as an exploratory multivariate visualisation of the organic acid profiles (Figure 9). The PCA was performed using standardised concentrations of eight organic acids. The first three principal components explained 80.3% of the total variance, with PC1 accounting for 41.7%, PC2 for 25.9% and PC3 for 12.7%.

The PCA score plots showed product-level differentiation according to organic acid composition. Birch chaga and hawthorn fruit syrups were separated from most other syrup products, mainly because of their high citric acid values in the present dataset. Quince fruit syrup was distinguished by its high malic acid content, while pine young shoot and spruce young shoot syrups were associated with higher quinic acid values. Rosebay willowherb flower and meadowsweet flower syrups formed a separate exploratory cluster characterised by detectable fumaric acid and higher succinic acid values compared with most other samples.

K-means clustering supported the exploratory differentiation observed in the PCA score space. Candidate cluster numbers from k = 2 to k = 5 were evaluated, and k = 4 was selected based on silhouette score and interpretability. The resulting clusters reflected differences in organic acid profiles rather than a strict separation according to raw material matrix group. Therefore, organic acid profiling provided useful product-level compositional information, but the clusters should be interpreted as exploratory groupings rather than definitive botanical classifications.

**Figure 10:**
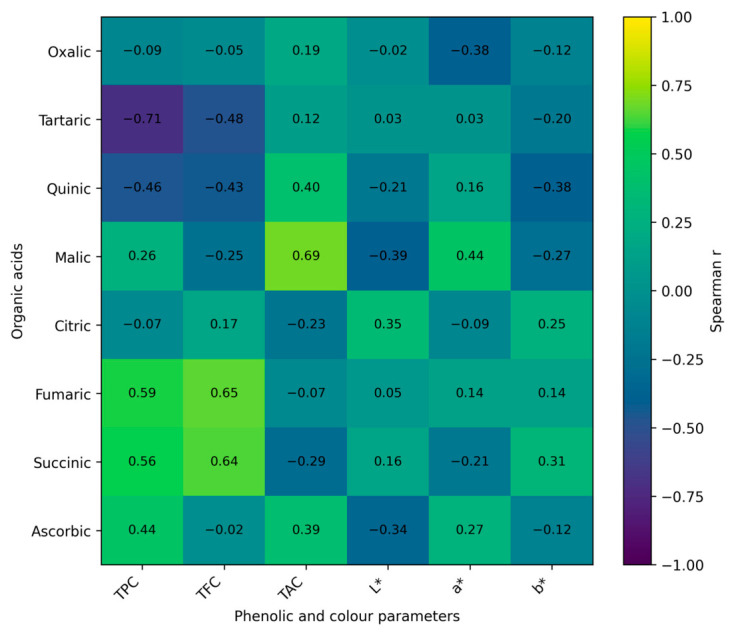
Spearman correlation heatmap between organic acids and phenolic/colour parameters in plant-derived syrups.

Spearman correlation analysis was used to visualise exploratory associations between individual organic acids and phenolic or colour-related parameters (Figure 10).

The Spearman heatmap showed that associations between organic acids and phenolic or colour-related parameters were variable and acid-specific. Malic acid showed a positive association with spectrophotometric TAC, while fumaric and succinic acids showed positive associations with TPC and TFC in the exploratory correlation matrix. Several organic acids showed weak associations with CIELAB colour parameters, indicating that organic acid composition alone did not consistently predict colour characteristics across the analysed syrup products. The heatmap was therefore interpreted as an exploratory pattern of product-level associations rather than confirmatory statistical evidence.

## 4. Discussion

The present research provides an integrated product-level characterisation of plant-derived syrup products by combining physicochemical parameters, phenolic and flavonoid indices, spectrophotometric TAC, smartphone-based CIELAB colour assessment and HPLC-DAD-determined organic acid composition. Compared with earlier studies on Latvian herbal and fruit syrups, which separately described antioxidant-related parameters or organic acid content [2], the present research combines these analytical groups within one comparative dataset. Therefore, the main contribution of this research lies in the integrated interpretation of final syrup products rather than in the confirmation of functional food effects.

pH and titratable acidity described complementary aspects of acidity in the analysed syrups. The moderate negative correlation between them indicated that products with higher titratable acidity generally showed lower pH, although the strength of the correlation showed that pH variation was not fully explained by titratable acidity, in line with differences in organic acid composition and matrix buffering. Total soluble solids, in contrast, showed no significant association with titratable acidity, because the °Brix of these products was largely determined by sucrose addition during preparation rather than by natural raw material constituents. °Brix, pH and titratable acidity are therefore complementary quality-related parameters and should not be treated as interchangeable indicators.

Phenolic and flavonoid indices differed markedly among products, and TPC and TFC were strongly and positively correlated in samples with quantifiable TFC, indicating that phenolic-rich syrups generally also contained more flavonoids. However, TPC should not be read as a measure of a single compound class, because the Folin–Ciocalteu assay responds to a broad range of reducing substances; the phenolic indices are therefore best interpreted as comparative rather than compound-specific values.

Colour was more closely related to spectrophotometric TAC than to total phenolic content. TPC alone was not a strong predictor of the CIELAB parameters—as expected given that phenolic compounds are chemically diverse and many are colourless or only weakly coloured. In contrast, TAC was significantly and negatively correlated with L* but only weakly with a*, showing that higher TAC was associated more with reduced lightness and a darker appearance than with increased redness. Because TAC was measured by a single-wavelength acidified ethanol method, these values represent comparative estimates rather than concentrations of individual anthocyanins.

The smartphone-based DiColorimetry approach provided standardised CIELAB descriptors that could be integrated with the compositional parameters, because the same imaging geometry, light box, background and region-of-interest procedure were applied to all samples. Nevertheless, the colour data should be interpreted as smartphone-based CIELAB analysis under the applied conditions and not as a full replacement for calibrated instrumental colorimetry.

The exploratory chemometric analyses reinforced these product-level patterns. In the phenolic and colour PCA, samples were separated mainly by colour-related variation and by phenolic and flavonoid indices, whereas the organic acid PCA and K-means clustering differentiated products by acid composition rather than by botanical matrix group; the Spearman analysis likewise showed only acid-specific and generally weak associations between individual organic acids and phenolic or colour parameters. Individual organic acids thus provided more detailed compositional information than total acidity alone, but all multivariate and correlation outputs are interpreted as exploratory product-level patterns rather than as definitive botanical or functional classifications or as evidence of causal relationships.

From an applied perspective, the dataset provides a compositional basis for selecting syrups for specific uses: higher titratable acidity where stronger acidity is desirable, higher TPC and TFC where phenolic-rich ingredients are sought, and higher TAC where a darker colour is preferred. However, these results do not demonstrate functional performance, sensory acceptance or health-related effects.

Several limitations should be considered. The research was based on raw materials collected during one vegetation season and on one prepared syrup batch per syrup type. Analytical measurements were performed as technical replicates; therefore, the reported variability reflects analytical repeatability rather than independent batch-to-batch variability. The results were expressed per 100 g of final syrup and should be interpreted as product-level characteristics rather than dry-matter-normalised raw material comparisons. In addition, TAC was determined using a spectrophotometric acidified ethanol method, and individual anthocyanins were not separated or identified. Sensory evaluation, antioxidant activity, storage stability, bioaccessibility and biological activity were not evaluated in the present research.

Overall, integrated profiling provided a broader characterisation of plant-derived syrup products than individual measurements alone, differentiating them according to compositional and visual quality-related attributes. These findings provide a basis for the further evaluation of selected syrups as potential natural food ingredients, but additional studies are required before functional food performance can be confirmed.

## 5. Conclusions

This study provided an integrated product-level characterisation of plant-derived syrups prepared from diverse botanical and traditionally used natural raw materials. The analysed syrup products differed markedly in their physicochemical parameters, phenolic and flavonoid indices, spectrophotometric TAC, smartphone-based CIELAB colour characteristics and HPLC-DAD-determined organic acid profiles. The results showed that syrup product characteristics were not described by a single parameter, but by the combined evaluation of acidity, soluble solids, phenolic and flavonoid indices, spectrophotometric TAC, colour parameters and individual organic acids.

Phenolic-rich syrups were not necessarily the most intensely coloured, while syrups with higher spectrophotometric TAC were more closely associated with darker colour expression than with consistently higher redness. Organic acid profiling provided additional differentiation among syrup products and supported the interpretation of product-level compositional patterns. Overall, the integrated analytical and exploratory chemometric approach used in this study provides a useful basis for the further evaluation and potential selection of plant-derived syrups as natural food ingredients according to targeted acidity, colour and compositional characteristics.

Future research should evaluate antioxidant activity, bioaccessibility, sensory properties, storage stability and biological activity, as well as the application potential of selected syrup products in food and beverage formulations.

## Figures and Tables

**Table 1:** Syrup types, botanical origin and harvesting locations of raw materials used for syrup preparation.

Type of Syrup/Botanical Origin (Raw Material Matrix Group)	Harvesting Location
Pine cone *Pinus sylvestris* L. (cones)	57°03′49.3″ N, 24°04′39.6″ E, WGS 84 Mangaļsala, Riga, Latvia
Rosehip fruit *Rosa canina* L. (fruits)	57°10′14.2″ N, 24°49′10.9″ E, WGS 84 Ragana, Sigulda Municipality, Latvia
Pine young shoot *Pinus sylvestris* L. (young shoots)	57°04′24.6″ N, 24°06′11.2″ E, WGS 84 Vecāķi, Riga, Latvia
Spruce young shoot *Picea abies* L. H.Karst. (young shoots)	57°15′25″ N, 24°52′45″ E, WGS 84 Inciems, Sigulda Municipality, Latvia
Dandelion flower *Taraxacum officinale* F.H.Wigg. (flowers)	57°15′25″ N, 24°52′45″ E, WGS 84 Inciems, Sigulda Municipality, Latvia
Rowan fruit *Sorbus aucuparia* L. (fruits)	57°15′25″ N, 24°52′45″ E, WGS 84 Inciems, Sigulda Municipality, Latvia
Rosebay willowherb flower *Chamaenerion angustifolium* L. Scop. (flowers)	57°15′25″ N, 24°52′45″ E, WGS 84 Inciems, Sigulda Municipality, Latvia
Hawthorn fruit *Crataegus* spp. (fruits)	57°10′14.2″ N, 24°49′10.9″ E, WGS 84 Ragana, Sigulda Municipality, Latvia
Apple fruit *Malus domestica* Borkh. (fruits)	57°15′25″ N, 24°52′45″ E, WGS 84 Inciems, Sigulda Municipality, Latvia
Meadowsweet flower *Filipendula ulmaria* L. Maxim. (flowers)	57°15′25″ N, 24°52′45″ E, WGS 84 Inciems, Sigulda Municipality, Latvia
Birch chaga *Inonotus obliquus* (Ach. ex Pers.) Pilát (chaga material)	57°15′25″ N, 24°52′45″ E, WGS 84 Inciems, Sigulda Municipality, Latvia
Lilac flower *Syringa vulgaris* L. (flowers)	57°15′25″ N, 24°52′45″ E, WGS 84 Inciems, Sigulda Municipality, Latvia
Quince fruit *Chaenomeles japonica* (Thunb.) Lindl. ex Spach (fruits)	57°15′25″ N, 24°52′45″ E, WGS 84 Inciems, Sigulda Municipality, Latvia
Guelder-rose fruit *Viburnum opulus* L. (fruits)	56°51′42.1″ N, 24°16′48.2″ E, WGS 84 Dārziņi neighbourhood, Riga, Latvia
Birch bark *Betula* spp. (bark)	57°15′25″ N, 24°52′45″ E, WGS 84 Inciems, Sigulda Municipality, Latvia

**Table 2:** Physicochemical characteristics of the analysed plant-derived syrups.

Sample	pH	TSS, °Brix	TA, mg CAE 100 g^−1^
Pine cone	3.68 ± 0.03	67.7 ± 0.4	438.53 ± 8.77
Rosehip fruit	3.71 ± 0.02	72.8 ± 0.3	249.63 ± 4.99
Pine young shoots	3.93 ± 0.04	71.2 ± 0.5	125.40 ± 2.51
Spruce young shoots	3.75 ± 0.03	69.9 ± 0.4	315.57 ± 6.31
Dandelion flower	3.79 ± 0.02	70.4 ± 0.3	174.81 ± 3.50
Rowan fruit	3.17 ± 0.04	64.7 ± 0.4	615.74 ± 12.31
Rosebay willowherb flower	3.90 ± 0.03	70.0 ± 0.6	306.98 ± 6.14
Hawthorn fruit	4.24 ± 0.05	60.2 ± 0.4	143.36 ± 2.87
Apple fruit	3.00 ± 0.02	62.4 ± 0.5	479.38 ± 9.59
Meadowsweet flower	3.82 ± 0.03	69.5 ± 0.5	277.36 ± 5.55
Birch chaga	4.60 ± 0.05	64.2 ± 0.6	49.15 ± 0.98
Lilac flower	5.40 ± 0.04	67.8 ± 0.4	165.43 ± 3.31
Quince fruit	2.60 ± 0.03	73.2 ± 0.3	1347.19 ± 26.94
Guelder-rose fruit	3.05 ± 0.02	59.4 ± 0.5	372.40 ± 7.45
Birch bark	2.60 ± 0.04	76.7 ± 0.6	259.94 ± 5.20

Values are expressed as mean ± SD of technical replicate measurements (*n* = 3); TSS—total soluble solids; TA—titratable acidity; CAE—citric acid equivalents.

**Table 3:** Phenolic and flavonoid contents, spectrophotometric total anthocyanin content and smartphone-based CIELAB colour parameters of the analysed plant-derived syrups.

Sample	Total Phenolic Content, mg GAE 100 g^−1^	Total Flavonoid Content, mg QE 100 g^−1^	Total Anthocyanin Content, mg 100 g^−1^	CIELAB		
				L*	a*	b*
Pine cone	360.62 ± 8.51	<1	3.03 ± 0.09	18.590 ± 0.350	4.631 ± 0.120	1.770 ± 0.080
Rosehip fruit	274.62 ± 7.24	4.93 ± 0.18	0.63 ± 0.03	38.874 ± 0.420	15.379 ± 0.280	36.473 ± 0.550
Pine young shoots	92.09 ± 3.10	3.25 ± 0.14	1.34 ± 0.05	52.145 ± 0.480	9.360 ± 0.200	42.694 ± 0.600
Spruce young shoots	178.44 ± 5.47	<1	2.98 ± 0.08	19.345 ± 0.360	10.662 ± 0.220	4.432 ± 0.120
Dandelion flower	83.50 ± 2.89	4.66 ± 0.17	0.13 ± 0.01	63.515 ± 0.550	3.623 ± 0.100	49.782 ± 0.650
Rowan fruit	180.14 ± 5.57	5.70 ± 0.20	1.65 ± 0.05	28.115 ± 0.380	22.209 ± 0.350	15.573 ± 0.320
Rosebay willowherb flower	446.36 ± 10.80	48.93 ± 1.45	0.97 ± 0.04	46.360 ± 0.450	10.758 ± 0.210	38.588 ± 0.560
Hawthorn fruit	150.35 ± 4.76	3.93 ± 0.15	0.95 ± 0.04	51.143 ± 0.470	11.444 ± 0.230	40.926 ± 0.580
Apple fruit	73.54 ± 2.54	3.31 ± 0.14	0.34 ± 0.02	49.701 ± 0.460	4.910 ± 0.130	41.303 ± 0.590
Meadowsweet flower	521.64 ± 12.54	62.34 ± 1.80	0.68 ± 0.03	44.694 ± 0.440	9.206 ± 0.190	39.650 ± 0.570
Birch chaga	89.50 ± 3.08	8.07 ± 0.25	0.68 ± 0.03	20.092 ± 0.360	6.365 ± 0.150	1.608 ± 0.070
Lilac flower	196.19 ± 5.91	8.79 ± 0.27	0.42 ± 0.02	68.171 ± 0.580	2.064 ± 0.090	51.584 ± 0.680
Quince fruit	239.16 ± 6.82	<1	1.19 ± 0.04	40.043 ± 0.430	8.137 ± 0.180	25.283 ± 0.430
Guelder-rose fruit	306.12 ± 7.80	6.87 ± 0.22	2.00 ± 0.06	30.089 ± 0.390	21.682 ± 0.340	16.912 ± 0.330
Birch bark	64.87 ± 2.26	3.34 ± 0.14	0.74 ± 0.03	60.101 ± 0.530	−2.142 ± 0.080	27.047 ± 0.450

Values are expressed as mean ± SD of technical replicate measurements (*n* = 3); TPC—total phenolic content; TFC—total flavonoid content; TAC—total anthocyanin content; GAE—gallic acid equivalent; QE—quercetin equivalent.

**Table 4:** Organic acid content of the analysed plant-derived syrups.

Syrup Type	Organic Acids, mg 100 g^−1^							
	Oxalic	Tartaric	Quinic	Malic	Citric	Fumaric	Succinic	Ascorbic
Pine cone	213 ± 3	n.d.	28 ± 1	271 ± 4	15 ± 1	n.d.	96 ± 1	55 ± 1
Rosehip fruit	95 ± 2	n.d.	20 ± 1	31 ± 1	139 ± 2	n.d.	23 ± 0	9 ± 0
Pine young shoots	91 ± 2	75 ± 2	479 ± 6	81 ± 2	328 ± 4	n.d.	n.d.	6 ± 0
Spruce young shoots	268 ± 4	79 ± 2	729 ± 8	130 ± 2	20 ± 1	n.d.	n.d.	6 ± 0
Dandelion flower	166 ± 3	5 ± 1	1 ± 0	66 ± 1	64 ± 2	n.d.	103 ± 1	n.d.
Rowan fruit	212 ± 3	47 ± 1	204 ± 3	544 ± 6	6 ± 0	n.d.	n.d.	n.d.
Rosebay willowherb flower	121 ± 2	3 ± 1	162 ± 3	64 ± 2	246 ± 3	1 ± 0	138 ± 1	2 ± 0
Hawthorn fruit	78 ± 2	178 ± 3	437 ± 5	126 ± 2	4849 ± 48	n.d.	n.d.	1 ± 0
Apple fruit	214 ± 3	45 ± 1	169 ± 3	49 ± 1	4 ± 0	n.d.	n.d.	n.d.
Meadowsweet flower	135 ± 2	n.d.	n.d.	19 ± 1	193 ± 3	1 ± 0	231 ± 2	1 ± 0
Birch chaga	n.d.	296 ± 4	758 ± 9	n.d.	11,140 ± 111	n.d.	n.d.	n.d.
Lilac flower	221 ± 3	7 ± 1	70 ± 2	23 ± 1	273 ± 4	n.d.	43 ± 0	n.d.
Quince fruit	222 ± 3	31 ± 1	308 ± 4	1208 ± 12	9 ± 0	n.d.	n.d.	n.d.
Guelder-rose fruit	212 ± 3	41 ± 1	183 ± 3	216 ± 3	17 ± 0	n.d.	n.d.	n.d.
Birch bark	239 ± 4	97 ± 2	264 ± 4	3 ± 0	169 ± 3	n.d.	n.d.	n.d.

Values are expressed as mean ± SD of technical replicate measurements (*n* = 3); n.d.—not detected; no identifiable peak corresponding to the retention time and UV spectral characteristics of the analytical standard was observed under the applied chromatographic conditions.

## Data Availability

The DiColorimetry Android application (version 1.2) used for smartphone-based colour analysis is available from Zenodo at https://doi.org/10.5281/zenodo.17819093. The software is distributed under a Custom Academic and Non-Commercial Licence.

## Supplementary Materials

The following supporting information can be downloaded at: https://www.mdpi.com/article/10.3390/foods15142500/s1, Figure S1: Screenshot of the DiColorimetry application interface showing smartphone-based image acquisition, region selection and real-time colorimetric data display.

## Abbreviations

The following abbreviations are used in this manuscript:

TPC	total phenolic content
TFC	total flavonoid content
TAC	total anthocyanin content
TSS	total soluble solids
TA	titratable acidity
PCA	principal component analysis
HPLC	high-performance liquid chromatography
CIELAB	CIE Lab* colour space
GAE	gallic acid equivalents
QE	quercetin equivalents
CAE	citric acid equivalents
SD	standard deviation.
