# Supplementary material for: Integrated Phytochemical, Anthocyanin, Organic Acid and Colour Profiling of Plant-Derived Syrups as Potential Natural Food Ingredients
Source: Foods. 2026 Jul 15;15(14):2500. doi: 10.3390/foods15142500 (PMC13409593; doi:10.3390/foods15142500)
Supplement: Supplementary file 1 [file foods-15-02500-s001.zip › foods-4412260-supplementary.pdf]

Supplementary Materials:

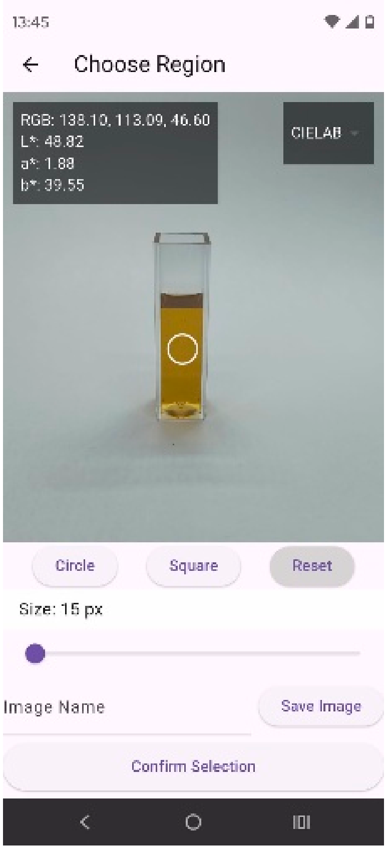

Figure S1. Screenshot of the DiColorimetry application interface showing smartphone-based image acquisition, region selection and real-time colorimetric data display.
